# Calcium oxalate crystals trigger epithelial-mesenchymal transition and carcinogenic features in renal cells: a crossroad between kidney stone disease and renal cancer

**DOI:** 10.1186/s40164-022-00320-y

**Published:** 2022-09-25

**Authors:** Paleerath Peerapen, Wanida Boonmark, Pattaranit Putpeerawit, Visith Thongboonkerd

**Affiliations:** Medical Proteomics Unit, Office for Research and Development, Faculty of Medicine Siriraj Hospital, Mahidol University, 6th Floor - SiMR Building, 2 Wanglang Road, Bangkoknoi, Bangkok, 10700 Thailand

**Keywords:** ARID1A, Carcinogenesis, EMT, PTEN, RCC, TPX2, VEGF, VHL

## Abstract

Increasing evidence of association between kidney stone disease (KSD) and renal cell carcinoma (RCC) has been reported. Nevertheless, mechanism underlying such association remained unknown. Herein, we investigated the effects of calcium oxalate monohydrate (COM), a major crystalline component causing KSD, on induction of carcinogenic features in non-cancerous renal cells. COM crystals induced morphological changes from epithelial to fibroblast-like spindle shape. Additionally, COM increased spindle index and mesenchymal markers (fibronectin and vimentin) but declined epithelial markers (E-cadherin and zonula occludens-1). Moreover, COM down-regulated *ARID1A*, a tumor suppressor gene recently reported to be reversely associated with RCC, at both mRNA and protein levels. COM also down-regulated other RCC-related tumor suppressor genes, *PTEN* and *VHL*, but up-regulated oncogene *TPX2*. Finally, COM enhanced invading capability, cell-aggregate formation, chemoresistance to cisplatin, and secretion of an angiogenic factor (VEGF). These data indicate that COM crystals trigger epithelial-mesenchymal transition (EMT) and several carcinogenic features in the non-cancerous renal cells. These mechanisms may explain and strengthen the association between KSD and RCC.

**Supplementary Information:**

The online version contains supplementary material available at 10.1186/s40164-022-00320-y.

To the Editor,

Kidney cancers are common around the globe accounting for 2% of diagnosed cancers with increasing incidence [1]. Among all types of kidney cancers, approximately 90% are renal cell carcinoma (RCC) [1]. Its incidence is twofold higher in men than women [1]. The precise etiology of RCC remains unclear; however, several genetic backgrounds, behaviors and environments are considered as the RCC risk factors. Increasing evidence of RCC has been reported in patients with kidney stone disease (KSD) [2–4]. On the other hand, several reports have shown intratumoral deposition of calcium oxalate (CaOx) crystals in RCC [5–7]. Therefore, KSD is now considered as another risk for RCC development. Nevertheless, the precise cellular and molecular mechanisms underlying this association have not been reported previously.

KSD is a common disease worldwide caused by intrarenal deposition of solid materials, comprising mainly CaOx monohydrate (COM) crystals [8]. COM crystals cause renal cell injury, induce reactive oxygen species (ROS) overproduction, and promote oxidative modifications of cellular proteins [8]. A previous study has revealed positive staining of oxidative DNA damage marker (8-OHdG) in renal tissue around the stone [9]. Interestingly, the oxidative DNA damage is also recognized to play crucial roles in initiation and progression of several cancers, including RCC [10, 11]. Similar to RCC, KSD is more common in males than females [12]. As KSD shares some disease backgrounds with RCC, we hypothesized that its crystalline component, COM, might be responsible for triggering carcinogenic features in non-cancerous renal cells.

Herein, we investigated the effects of COM crystals on induction of carcinogenic features in non-cancerous renal cells. Several assays were performed to investigate these carcinogenic features, including morphological changes, spindle index, epithelial-mesenchymal transition (EMT), expression of RCC-related tumor suppressor genes (*ARID1A*, *PTEN*, and *VHL*) and oncogene (*TPX2*) (http://portal.gdc.cancer.gov/), cell invasion ability, cell-aggregate formation, chemoresistance, secretion of an angiogenic factor (vascular endothelial growth factor or VEGF) (see details in Additional file 1).

COM crystals induced morphological changes from epithelial to fibroblast-like spindle shape and increased spindle index (Fig. 1A, B). Without significant toxic effects (Fig. 1C, D), COM suppressed epithelial markers (E-cadherin and zonula occludens-1, ZO-1) (Fig. 1E–H) but enhanced mesenchymal markers (fibronectin and vimentin) (Fig. 1I–L). Moreover, COM down-regulated *ARID1A*, a tumor suppressor gene recently reported to be reversely associated with RCC, at both mRNA and protein levels (Fig. 2A–C). COM also down-regulated other RCC-related tumor suppressor genes, *PTEN* and *VHL*, but up-regulated the oncogene *TPX2* (Fig. 2D–F). Finally, COM enhanced invading capability (Fig. 2G, H), cell-aggregate formation (Fig. 2I, J), chemoresistance to cisplatin (Fig. 2K, L), and secretion of the angiogenic factor VEGF (Fig. 2M).

**Fig. 1:**
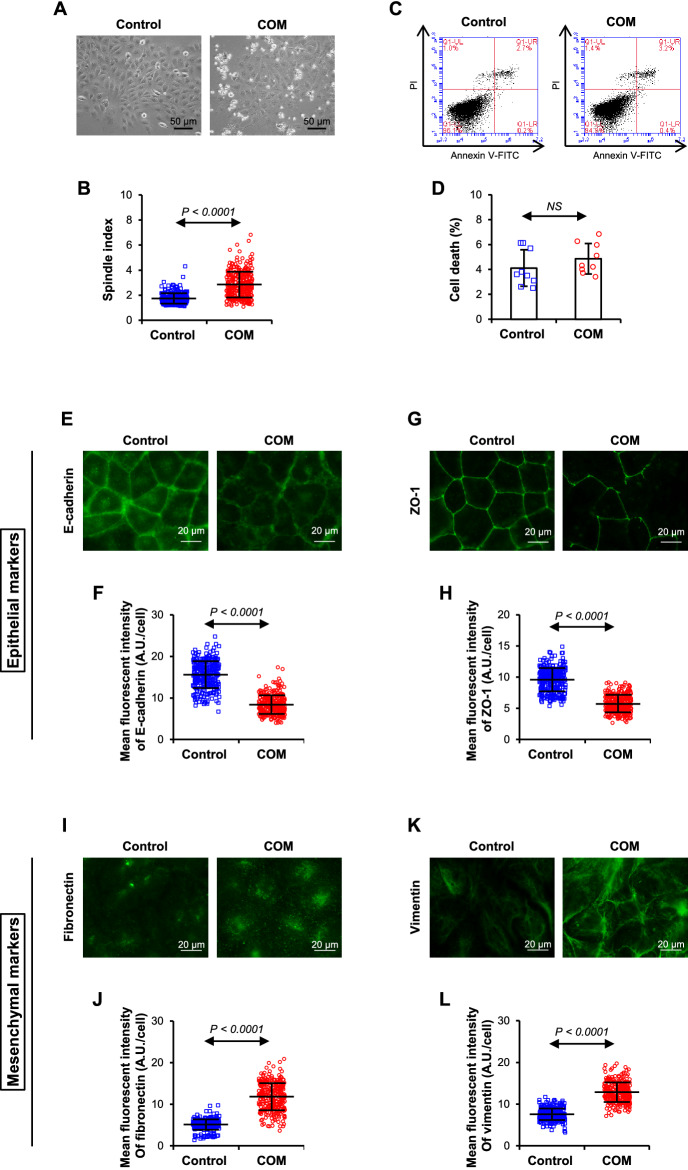
COM crystals trigger epithelial-mesenchymal transition (EMT) in renal cells. **A** Cell morphology. **B** Spindle index. **C**, **D** Flow cytometry with annexin V/propidium iodide stainings. **E–H** Epithelial markers (E-cadherin and ZO-1). **I–L** Mesenchymal markers (fibronectin and vimentin). All quantitative data are presented as mean ± SD derived from three independent experiments using different biological samples. *A.U.* arbitrary unit

**Fig. 2:**
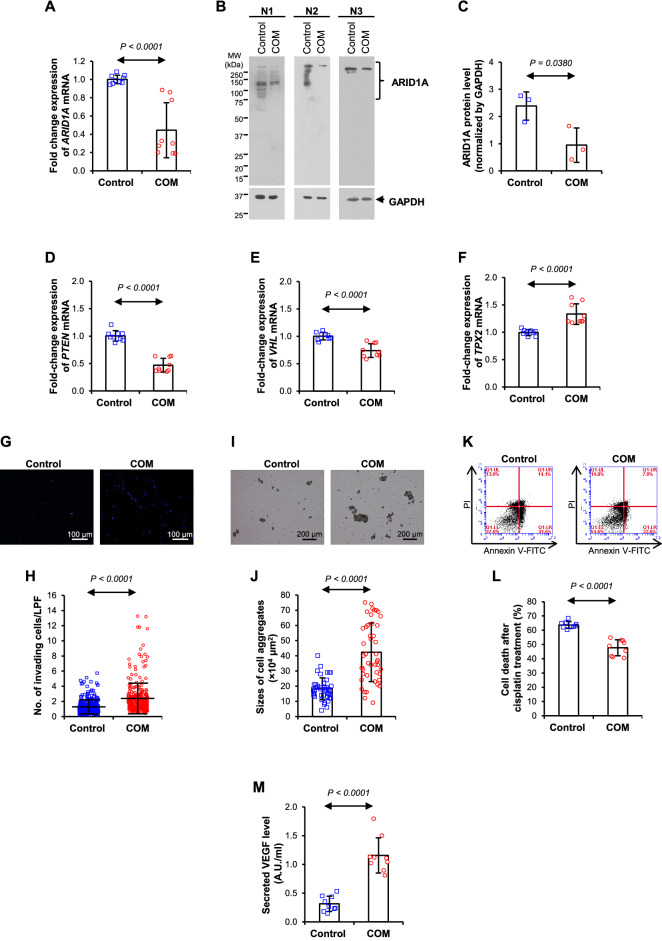
COM crystals trigger carcinogenic features of renal cells. **A**–**C** *ARID1A* mRNA and protein levels (GAPDH served as a loading control). **D–F** mRNA levels of *PTEN*, *VHL*, and *TPX2*, respectively. **G**, **H** Cell invasion assay. **I**, **J** Cell-aggregate formation (hanging-drop) assay. **K**, **L** Chemoresistance assay. **M** Level of VEGF secretion. All quantitative data are presented as mean ± SD derived from three independent experiments using different biological samples. *A.U.* arbitrary unit

EMT is the process in which epithelial cells structurally and functionally change to mesenchymal phenotype. This process is involved in several biological phenomena under both physiologic and pathogenic conditions, including embryogenesis, wound healing, tissue fibrogenesis, and carcinogenesis. It’s not unexpected that COM caused EMT. As EMT is one of the carcinogenic features, we hypothesized that COM crystals might induce other carcinogenic effects on the non-cancerous renal cells and may serve as a crossroad between KSD and RCC. Our hypothesis was supported by several assays to confirm such phenomenon. Together with down-regulations of the RCC-related tumor suppressor genes (*ARID1A*, *PTEN*, and *VHL*) and up-regulation of the oncogene (*TPX2*), other functional assays confirmed that COM crystals trigger several of the carcinogenic features, including invading capability, cell-aggregate formation, and chemoresistance to cisplatin. Finally, the COM-treated cells secrete greater level of an angiogenic factor, VEGF.

Our previous study has demonstrated that COM crystals trigger oxidative stress and induces oxidatively modified proteins in renal cells via ROS overproduction [8]. Therefore, COM may induce the oxidative DNA damage via this mechanism, thereby enhancing the carcinogenic features in non-cancerous renal cells. However, the precise mechanisms leading to COM-induced alterations in RCC-related tumor suppressor genes and oncogenes and increase of angiogenic factor remain unknow and deserve further investigations that may lead to better understanding of carcinogenesis induced by COM crystals.

Taken together, these data indicate that COM crystals trigger EMT and several of carcinogenic features in the non-cancerous renal cells. These mechanisms may explain and strengthen the association between KSD and RCC.

## Supplementary Information

**Additional file 1.** Supplementary methods.

## Data Availability

All data generated or analyzed during this study are included in this published article and are also available from the corresponding author on reasonable request.

## References

[1] Siegel RL, Miller KD, Fuchs HE, Jemal A (2021). Cancer statistics, 2021. CA Cancer J Clin.

[2] Cheungpasitporn W, Thongprayoon C, O'Corragain OA, Edmonds PJ, Ungprasert P, Kittanamongkolchai W (2015). The risk of kidney cancer in patients with kidney stones: a systematic review and meta-analysis. QJM.

[3] van de Pol JAA, van den Brandt PA, Schouten LJ (2019). Kidney stones and the risk of renal cell carcinoma and upper tract urothelial carcinoma: the Netherlands cohort study. Br J Cancer.

[4] Chung SD, Liu SP, Lin HC (2013). A population-based study on the association between urinary calculi and kidney cancer. Can Urol Assoc J.

[5] Rioux-Leclercq NC, Epstein JI (2003). Renal cell carcinoma with intratumoral calcium oxalate crystal deposition in patients with acquired cystic disease of the kidney. Arch Pathol Lab Med.

[6] Sule N, Yakupoglu U, Shen SS, Krishnan B, Yang G, Lerner S (2005). Calcium oxalate deposition in renal cell carcinoma associated with acquired cystic kidney disease: a comprehensive study. Am J Surg Pathol.

[7] Enoki Y, Katoh G, Okabe H, Yanagisawa A (2010). Clinicopathological features and CD57 expression in renal cell carcinoma in acquired cystic disease of the kidneys: with special emphasis on a relation to the duration of haemodialysis, the degree of calcium oxalate deposition, histological type, and possible tumorigenesis. Histopathology.

[8] Vinaiphat A, Aluksanasuwan S, Manissorn J, Sutthimethakorn S, Thongboonkerd V (2017). Response of renal tubular cells to differential types and doses of calcium oxalate crystals: Integrative proteome network analysis and functional investigations. Proteomics.

[9] Kittikowit W, Waiwijit U, Boonla C, Ruangvejvorachai P, Pimratana C, Predanon C (2014). Increased oxidative DNA damage seen in renal biopsies adjacent stones in patients with nephrolithiasis. Urolithiasis.

[10] Srinivas US, Tan BWQ, Vellayappan BA, Jeyasekharan AD (2019). ROS and the DNA damage response in cancer. Redox Biol.

[11] Guo E, Wu C, Ming J, Zhang W, Zhang L, Hu G (2020). The clinical significance of DNA damage repair signatures in clear cell renal cell carcinoma. Front Genet.

[12] Gillams K, Juliebo-Jones P, Juliebo SO, Somani BK (2021). Gender differences in kidney stone disease (KSD): findings from a systematic review. Curr Urol Rep.

