# Supplementary material for: Calcium oxalate crystals trigger epithelial-mesenchymal transition and carcinogenic features in renal cells: a crossroad between kidney stone disease and renal cancer
Source: Exp Hematol Oncol. 2022 Sep 25;11:62. doi: 10.1186/s40164-022-00320-y (PMC9509655; doi:10.1186/s40164-022-00320-y)
Supplement: Supplementary file 1 — Additional file 1. Supplementary methods. [file 40164_2022_320_MOESM1_ESM.pdf]

# SUPPLEMENTARY METHODS

## Calcium oxalate crystals trigger epithelial-mesenchymal transition and carcinogenic features in renal cells: A crossroad between kidney stone disease and renal cancer

Paleerath Peerapen, Wanida Boonmark, Pattaranit Putpeerawit, and Visith Thongboonkerd*
(*Correspondence to: thongboonkerd@dr.com (or) vthongbo@yahoo.com)

### COM crystallization and harvest

COM crystals were generated using a well-established protocol [1, 2] with slight modifications. Briefly, calcium chloride ($CaCl_2{\cdot}2H_2O$) (Merck; Branchburg, NJ) (at 10 mM) and sodium oxalate ($Na_2C_2O_4$) (Sigma-Aldrich; St. Louis, MO) (at 1 mM) solutions were prepared in a crystallization buffer (pH 7.4) containing 10 mM Tris (Affymetrix inc.; Cleveland, OH) and 90 mM sodium chloride (NaCl) (Bio Basic; Toronto, Canada). Thereafter, 10 mM $CaCl_2{\cdot}2H_2O$ was mixed (1:1) with 1 mM $Na_2C_2O_4$ and incubated overnight at 25°C. The crystals were harvested by 5-min centrifugation at 2,000 ×*g* and washed with methanol three times, followed by 5-min centrifugation at 2,000 ×*g*. Finally, the crystals were air-dried overnight. Typical morphology of the COM crystal was validated, and the crystals were subjected to UV radiation for 30 min to decontaminate before use.

### Non-cancerous renal cell culture and COM crystal intervention

Cell culture and COM crystal intervention were performed as described previously [3, 4]. MDCK cells (ATCC; Manassas, VA) (approximately $4 \times 10^4$ cells/well) were plated and grown in each well of 6-well plate (Corning Inc.; Corning, NY) containing Eagle's minimum essential medium (MEM) (Gibco; Grand Island, NY) supplemented with 10% fetal bovine serum (FBS) (Gibco), 60 U/ml penicillin G (Sigma-Aldrich) and 60 µg/ml streptomycin (Sigma-Aldrich). The cells were maintained in a humidified incubator at 37°C with 5% $CO_2$ overnight, and the medium was discarded. Thereafter, the cells were further incubated in MEM without (control) or with non-toxic dose (100 µg/ml) of COM crystals for 24 h (for almost all of experiments, except only for mRNA measurement, which required only 8-h incubation) and then subjected to investigations as follows.

### Morphological examination and spindle index measurement

After 24-h incubation with or without COM crystals, cell morphology was examined and imaged under the Nikon Eclipse Ti-S inverted phase-contrast light microscope (Nikon; Tokyo, Japan). Cell boundary was manually determined, whereas length and width of each cell were automatically measured using NIS-Elements D V.4.11 software (Nikon). Spindle index [5, 6] was calculated from at least 100 cells in ≥ 10 random high-power fields (HPFs) per each biological sample using the following formula:

*Formula 1*: Spindle index = Length of each cell / Width of each cell

### Flow cytometry with annexin V/propidium iodide stainings

Cell death assay was performed using fluorescein isothiocyanate (FITC)-conjugated annexin V (BD Biosciences; San Jose, CA) and propidium iodide (BD Biosciences) stainings as described previously [7, 8]. After 24-h incubation with or without COM crystals, detached cells were combined with floating cells, pelleted by centrifugation at 500 ×*g* and 4°C for 5 min, and washed twice with ice-cold PBS. Thereafter, the cells were incubated with FITC-conjugated annexin V diluted in annexin V binding buffer (BD Biosciences) at 25°C in the dark for 15 min. Subsequently, propidium iodide at the final concentration of 0.2 µg/ml was

added, and the stained cells were analyzed by using BD Accuri C6 flow cytometer (BD Biosciences). Three technical replicates were done per each biological sample.

**Immunofluorescence cellular staining**

The cells were maintained and treated as aforementioned but on coverslip in each well. After 24-h incubation with or without COM crystals, the cells were subjected to immunofluorescence stainings as previously reported [9, 10]. Briefly, the cells were fixed with 4% paraformaldehyde at 25°C for 15 min followed by permeabilization with 0.1% Triton X-100 in PBS at 25°C for 15 min. Non-specific bindings were blocked with 1% bovine serum albumin (BSA) (Sigma-Aldrich) in PBS at 25°C for 30 min. The cells were then incubated with rat monoclonal anti-E-cadherin (Santa Cruz Biotechnology; Santa Cruz, CA,), mouse monoclonal anti-fibronectin (Santa Cruz Biotechnology), mouse monoclonal anti-vimentin (Santa Cruz Biotechnology), or mouse monoclonal anti-ZO-1 antibody (Invitrogen; Carlsbad, CA) (all were diluted 1:50 in 1% BSA/PBS) at 37°C for 1 h. After three washes with PBS, the cells were incubated with Alexa Flour 488-conjugated anti-rat or anti-mouse IgG (Invitrogen) (1:500 in 1% BSA/PBS containing 1:1,000 Hoechst dye) at 37°C for 1 h. After mounting with slide, the cells were examined and imaged under a fluorescence microscope (Eclipse 80i) (Nikon). Quantitative intensity data were measured from at least 100 cells in ≥ 10 random HPFs per each biological sample using NIS-Elements D V.4.11 (Nikon).

**Quantitative RT-PCR (qRT-PCR)**

After 8-h incubation with or without COM crystals, culture medium was removed and the cells were washed with PBS. TRIzol reagent (Invitrogen) was added into each well for total RNA extraction. The solution was collected and RNA purification was performed using Direct-zol RNA MiniPrep (Zymo Research; Irvine, CA). cDNA was synthesized from an equal amount of purified total RNA via reverse transcription using SuperScript III reverse transcriptase (Thermo Fisher Scientific; Waltham, MA) and random primer. To assess mRNA expression levels of *ARID1A*, *PTEN*, *VHL and TPX2*, iTaq universal SYBR Green Supermix (Bio-Rad; Hercules, CA) was applied for qRT-PCR using specific primers as follows.

Summary of all primers used in this study.

| **Gene** | **Primer (5'→ 3')** | **Product size (bp)** |
|---|---|---|
| *ARID1A* | **Forward:** CCCCTCAATGACCTCCAGTA<br>**Reverse:** CTGGAAATCCCTGATGTGCT | 159 |
| *PTEN* | **Forward:** GCAGAAAGACTTGAAGGCG<br>**Reverse:** GCTGTGGTGGATTATGGTC | 178 |
| *VHL* | **Forward:** CAATGTTGATGGACAGCC<br>**Reverse:** CATTCGGGTGGTCTTCC | 161 |
| *TPX2* | **Forward:** GACTTCCACTTCCGCAC<br>**Reverse:** CCTTGGGACAGGTTGAAAG | 161 |
| *GAPDH* | **Forward:** GCCAAGAGGGTCATCATCTC<br>**Reverse:** GCATGGACTGTGGTCATG | 194 |

A total of 40 cycles for amplification of qRT-PCR reactions (denaturation at 95°C for 5 s, annealing/extension at 58-60°C for 30 s) were performed in CFX Connect Real-Time PCR Detection System (Bio-Rad). The housekeeping gene, *GAPDH*, served as an internal control to normalize level of the target gene. Fold-change expression of each gene was

calculated from technical triplicate per each biological sample using the comparative $2^{-\Delta\Delta Ct}$ method as follows:

*Formula 2*: $\Delta Ct = Ct_{\text{Target gene}} - Ct_{GAPDH}$

*Formula 3*: $\Delta\Delta Ct = \Delta Ct_{COM} - \Delta Ct_{Control}$

*Formula 4*: Fold-change expression = $2^{-\Delta\Delta Ct}$

Where Ct (cycle threshold) = the cycle numbers, at which the fluorescence signal exceeds background signal (or detection threshold).

**Western blotting**

After 24-h incubation with or without COM crystals, cellular proteins were solubilized by Laemmli's buffer, and protein concentrations were measured by Bradford's method. Western blotting was performed as reported previously [11, 12]. Proteins were resolved by 12% SDS-PAGE with equal loading (50 µg/lane) and then transferred onto a nitrocellulose membrane. After blocking non-specific binders with 5% skim-milk/PBS for 1 h, the membrane was incubated overnight at 4°C with mouse monoclonal anti-ARID1A (Santa Cruz Biotechnology) or anti-GAPDH antibody (Santa Cruz Biotechnology) (1:1,000 in 1% skim milk/PBS). After washing with PBS three times, the membrane was incubated with rabbit anti-mouse IgG conjugated with horseradish peroxidase (1:20,000 in 1% skim-milk/PBS) (Sigma-Aldrich) at 25°C for 1 h. The immunoreactive protein bands were visualized by SuperSignal West Pico chemiluminescence substrate (Pierce Biotechnology, Inc.; Rockford, IL) and quantified by using ImageQuant TL software (GE Healthcare; Uppsala, Sweden).

**Cell invasion assay**

Cell invasion assay was performed as described previously [13, 14]. Briefly, membrane insert of Transwell culture plate (5-µm pore size, 0.33-$\mu m^2$ surface area) (Corning Costar; Cambridge, MA) was pre-coated with Matrigel (BD Biosciences) at 37°C overnight. After 24-h incubation with or without COM crystals, the cells were detached and resuspended in non-supplemented MEM at a density of $1\times10^6$ cells/ml. Approximately $2\times10^5$ cells (200 µl) were seeded into upper chamber of each well, whereas the lower chamber was filled with 10% FBS-supplemented MEM. After 24-h incubation at 37°C in a humidified incubator with 5% $CO_2$ for 24 h, the cells remained above membrane insert were removed, whereas those appeared under the membrane insert were fixed with 4% (w/v) paraformaldehyde/PBS for 15 min and stained with Hoechst dye (1:2,000 in PBS) at 25°C for 10 min. The invading cells were imaged under a fluorescence microscope (Eclipse 80i) (Nikon). Number of the invading cells was counted from at least 15 random low-power fields (LPFs) per each biological sample.

**Cell-aggregate formation (hanging-drop) assay**

Hanging drop assay was performed as described previously [13, 14]. After 24-h incubation with or without COM crystals, the cells were detached and resuspend in 10% FBS-supplemented MEM at a density of $2.5\times10^5$ cells/ml. For each sample, a total of 10 drops of cell suspension (approximately 20 µl/drop containing $5\times10^3$ cells) was spotted on the inner side of upper lid, which was then placed on top of 100-mm culture dish containing 5-ml of 10% FBS-supplemented MEM. After 24-h incubation at 37°C in a humidified incubator with 5% $CO_2$, the cells were harvested and pipetted up and down several times. Cell-aggregate formation was imaged under the Eclipse Ti-S inverted phase-contrast microscope (Nikon) and analyzed by using the NIS-Elements D software version 4.11 (Nikon). The cell-aggregate size was measured from at least 100 individual aggregates per each biological sample.

**Chemoresistance assay**

Cisplatin (Biochem Pharmaceutical Industries Ltd., Daman, India) was used as a chemotherapeutic drug against cancerous cells. After 24-h incubation with or without COM crystals, the cells were treated with 50 µM cisplatin for the next 24 h. Cell death was then quantified by flow cytometry using annexin V/propidium iodide stainings as described above.

**ELISA**

ELISA was performed as previously described [15, 16]. After 24-h incubation with or without COM crystals, the cells were further incubated in 2 ml non-supplemented MEM for 24 h. The serum-free medium was collected, desalted by dialysis against deionized water, and lyophilized. The protein powder was re-solubilized in 0.05 ml of coating buffer (30 mM $NaHCO_3$ and 15 mM $Na_2CO_3$; pH 9.4). The secreted protein sample with an equal amount (2.5 µg/well) was added into each well of 96-well ELISA plate (Nunc; Roskilde, Denmark) and incubated at 4°C for 24 h. After discarding the coating buffer, each well was washed with 0.05% Tween-20/PBS five times and incubated with 1% BSA/PBS at 25°C for 2 h to block non-specific binders. After other five washes with 0.05% Tween-20/PBS, each well was incubated with mouse monoclonal anti-VEGF antibody (Santa Cruz Biotechnology) (1:1,000 in 0.1% BSA/PBS) at 25°C for 2 h. After other five washes, each well was incubated with rabbit anti-mouse IgG conjugated with horseradish peroxidase (Sigma-Aldrich) (1:20,000 in 0.1% BSA/PBS) at 25°C for 1 h in the dark. After the final five washes, the sample was incubated with 100 µl of 1.5 mM ortho-phenylenediamine dihydrochloride (Sigma-Aldrich) in 35 mM citric acid (Bio-Basic; Markham, Canada) and 0.012% $H_2O_2$ (Fisher Scientific; Loughborough, UK) (pH 5.5) in the dark for 15 min. Finally, the reaction was stopped by adding 50 µl of 2 M $H_2SO_4$. Absorbance of each sample was measured at λ492 nm using an ELISA microplate reader (EZRead 400, Biochrom Ltd.; Cambridge, UK). VEGF level was calculated from three technical replicates per each biological sample as follows:

*Formula 5*: Re-solubilized factor
= Starting medium volume (2 ml) / Resuspension volume (0.05 ml)

*Formula 6*: Secreted VEGF level (A.U./ml)
= $OD_{492}$ (A.U.) / [Loaded volume (ml) × Re-solubilized factor]

A.U. stands for arbitrary unit.

**Statistical analysis**

All quantitative data were obtained from three independent experiments using different biological samples and are presented as mean ± SD. Unpaired Student's t-test was performed for comparisons. The statistically significant differences were set at $P < 0.05$.

**References**

1. Thongboonkerd V, Semangoen T, Chutipongtanate S. Factors determining types and morphologies of calcium oxalate crystals: Molar concentrations, buffering, pH, stirring and temperature. Clin Chim Acta. 2006;367(1-2):120-31. doi: 10.1016/j.cca.2005.11.033.

2. Thongboonkerd V, Chutipongtanate S, Semangoen T, Malasit P. Urinary trefoil factor 1 is a novel potent inhibitor of calcium oxalate crystal growth and aggregation. J Urol. 2008;179(4):1615-9. doi: 10.1016/j.juro.2007.11.041.

3. Vinaiphat A, Aluksanasuwan S, Manissorn J, Sutthimethakorn S, Thongboonkerd V. Response of renal tubular cells to differential types and doses of calcium oxalate crystals: Integrative proteome network analysis and functional investigations. Proteomics. 2017;17(15-16):1700192. doi: 10.1002/pmic.201700192.

4. Semangoen T, Sinchaikul S, Chen ST, Thongboonkerd V. Proteomic analysis of altered proteins in distal renal tubular cells in response to calcium oxalate monohydrate crystal adhesion: Implications for kidney stone disease. Proteomics Clin Appl. 2008;2(7-8):1099-109. doi: 10.1002/prca.200780136.

5. Kanlaya R, Peerapen P, Nilnumkhum A, Plumworasawat S, Sueksakit K, Thongboonkerd V. Epigallocatechin-3-gallate prevents TGF-beta1-induced epithelial-mesenchymal transition and fibrotic changes of renal cells via GSK-3beta/beta-catenin/Snail1 and Nrf2 pathways. J Nutr Biochem. 2020;76:108266. doi: 10.1016/j.jnutbio.2019.108266.

6. Thanomkitti K, Fong-ngern K, Sueksakit K, Thuangtong R, Thongboonkerd V. Molecular functional analyses revealed essential roles of HSP90 and lamin A/C in growth, migration, and self-aggregation of dermal papilla cells. Cell Death Discov. 2018;4:53. doi: 10.1038/s41420-018-0053-6.

7. Sueksakit K, Thongboonkerd V. Protective effects of finasteride against testosterone-induced calcium oxalate crystallization and crystal-cell adhesion. J Biol Inorg Chem. 2019;24(7):973-83. doi: 10.1007/s00775-019-01692-z.

8. Chaiyarit S, Thongboonkerd V. Changes in mitochondrial proteome of renal tubular cells induced by calcium oxalate monohydrate crystal adhesion and internalization are related to mitochondrial dysfunction. J Proteome Res. 2012;11(6):3269-80. doi: 10.1021/pr300018c.

9. Kapincharanon C, Thongboonkerd V. K(+) deficiency caused defects in renal tubular cell proliferation, oxidative stress response, tissue repair and tight junction integrity, but enhanced energy production, proteasome function and cellular K(+) uptake. Cell Adh Migr. 2018;12(3):247-58. doi: 10.1080/19336918.2017.1356554.

10. Fong-ngern K, Vinaiphat A, Thongboonkerd V. Microvillar injury in renal tubular epithelial cells induced by calcium oxalate crystal and the protective role of epigallocatechin-3-gallate. FASEB J. 2017;31(1):120-31. doi: 10.1096/fj.201600543R.

11. Fong-ngern K, Ausakunpipat N, Singhto N, Sueksakit K, Thongboonkerd V. Prolonged K(+) deficiency increases intracellular ATP, cell cycle arrest and cell death in renal tubular cells. Metabolism. 2017;74:47-61. doi: 10.1016/j.metabol.2016.12.014.

12. Aluksanasuwan S, Sueksakit K, Fong-ngern K, Thongboonkerd V. Role of HSP60 (HSPD1) in diabetes-induced renal tubular dysfunction: regulation of intracellular protein aggregation, ATP production, and oxidative stress. FASEB J. 2017;31(5):2157-67. doi: 10.1096/fj.201600910RR.

13. Somsuan K, Peerapen P, Boonmark W, Plumworasawat S, Samol R, Sakulsak N, et al. ARID1A knockdown triggers epithelial-mesenchymal transition and carcinogenesis features of renal cells: role in renal cell carcinoma. FASEB J. 2019;33(11):12226-39. doi: 10.1096/fj.201802720RR.

14. Peerapen P, Sueksakit K, Boonmark W, Yoodee S, Thongboonkerd V. ARID1A knockdown enhances carcinogenesis features and aggressiveness of Caco-2 colon cancer cells: An in vitro cellular mechanism study. J Cancer. 2022;13(2):373-84. doi: 10.7150/jca.65511.

15. Yoodee S, Peerapen P, Plumworasawat S, Thongboonkerd V. ARID1A knockdown in human endothelial cells directly induces angiogenesis by regulating angiopoietin-2 secretion and endothelial cell activity. Int J Biol Macromol. 2021;180:1-13. doi: 10.1016/j.ijbiomac.2021.02.218.

16. Gallemit PEM, Yoodee S, Malaitad T, Thongboonkerd V. Epigallocatechin-3-gallate plays more predominant roles than caffeine for inducing actin-crosslinking, ubiquitin/proteasome activity and glycolysis, and suppressing angiogenesis features of human endothelial cells. Biomed Pharmacother. 2021;141:111837. doi: 10.1016/j.biopha.2021.111837.
